# Efficient and Explainable Virtual Screening of Molecules through Fingerprint-Generating Networks Integrated with Artificial Neural Networks

**DOI:** 10.1021/acsomega.4c10289

**Published:** 2025-01-28

**Authors:** Rivaaj Monsia, Sudeep Bhattacharyya

**Affiliations:** Department of Chemistry and Biochemistry, University of Wisconsin—Eau Claire, Eau Claire, Wisconsin 54701, United States

## Abstract

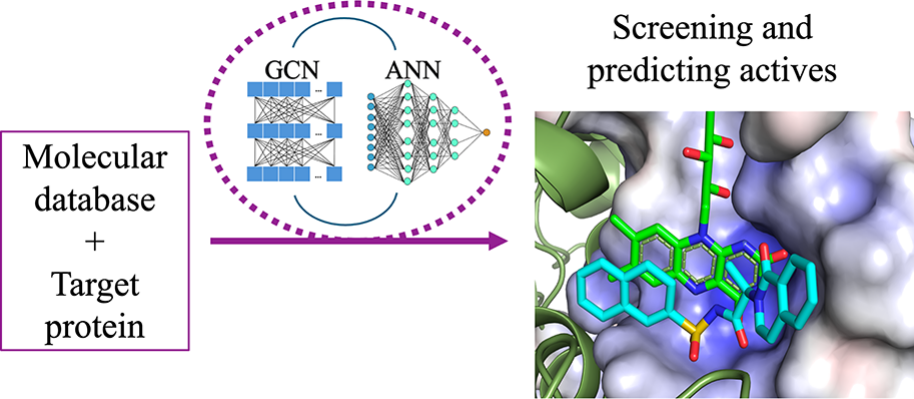

A machine learning-based drug screening technique has been developed and optimized using a novel, stitched neural network architecture with trainable, graph convolution-based fingerprints as a base into an artificial neural network. The architecture is efficient, explainable, and performant as a tool for the binary classification of ligands based on a user-chosen docking score threshold. Assessment using two standardized virtual screening databases substantiated the architecture’s ability to learn molecular features and substructures and predict ligand classes based on binding affinity values more effectively than similar contemporary counterparts. Furthermore, to highlight the architecture’s utility to groups and laboratories with varying resources, experiments were carried out using randomly sampled small molecules from the ZINC database and their computational docking scores against six drug-design relevant proteins. This new architecture proved to be more efficient in screening molecules that less favorably bind to a specific target thereby retaining top-hit molecules. Compared to similar protocols developed using Morgan fingerprints, the neural fingerprint-based model shows superiority in retaining the best ligands while filtering molecules at a higher relative rate. Lastly, the explainability of the model was investigated; it was revealed that the model accurately emphasized important chemical substructures and atoms through the intermediate fingerprint, which, in turn, contributed heavily to the ultimate prediction of a ligand as binding tightly to a certain protein.

## Introduction

Computational chemistry is essential to probing complex chemical systems and plays a critical role in computer-aided drug design.^1,2^ With the availability of large data sets, machine learning and artificial intelligence-embedded computational chemistry tools are becoming increasingly popular^3−7^ and hold great promise to shed light on several challenging fields of chemistry including structure-guided drug design.^2,8−14^ Drug discovery is a multistep process; several estimates characterize this process as taking on average, 12–18 years and around 2.6 billion US$ for a drug to progress from a laboratory discovery to a patient’s bedside. Novel machine-learning applications are being explored to expedite certain parts of the drug design timeline, namely compound screening.^2,8−19^ This multidisciplinary research area dominated by computational chemistry, data science, and machine learning seems to hold large untapped resources for structure-guided drug discovery that could alter the landscape of healthcare.^8,13,15^

For over a decade, computational chemistry has been employed in this lab in elucidating the chemistry of enzymes that are drug targets.^20−29^ Using classical and quantum physics-based models, these studies explored intermolecular interactions,^20,22,24^ receptor–ligand complexes,^21−23^ redox processes,^25−28^ and enzyme catalysis.^21,23,25^ However, these physics-based approaches failed to capture the information on selective molecular recognition and translate that to a rigorous inhibitor design. This is because a study of selective inhibition requires a molecular docking process that necessitates the representation of a part of the enzyme’s active site and each small molecule in a database and hence is computationally expensive. Consequently, they were not very effective in molecular screening of a large-molecular database.^30^ This prompted us to explore artificial intelligence-embedded computational screening techniques.

A single molecule can be thought of as a sentence composed of atoms or substructures as words.^31^ These substructures are combined in different patterns generating various molecular structures. Thus, encoders mimicking those used in natural language processing appear to hold great promise in learning the physics of molecular interactions and deciphering molecular substructures recognized by an enzyme.^32^ In particular, many such efforts have focused on abstract features of molecules via self-supervised learning, which is then used to train for predicting specific properties.^31,33−36^ In the present study, we integrated a neural network architecture with trainable, graph convolution-based fingerprints with the hope of finding an efficient inhibitor screening technique that can accelerate the process of exploring novel ligand chemical space.

## Theory and Methods

Computations were carried out on the hybrid GPU-CPU BOSE Cluster located at the Blugold Center for High-Performance Computing, UW-Eau Claire. Inhibitor-bound protein coordinates were obtained from Protein Data Bank.^37^ VMD was used for visualization and molecular editing.^38^ For computing molecular properties, the Open Babel codes^39^ were used to interchange molecular structures in various formats. Molecular docking on specific target proteins was carried out using AutoDockFR.^40^ Reading and writing of machine-readable molecular substructures were done using RDKit.^41^ The precision, recall, and receiver operating characteristics were calculated using the scikit-learn (version 1.4) library.^42^ The technical implementation of neural network architecture was done in PyTorch.^43^

### Overall Framework of the Study

**Scheme 1:**
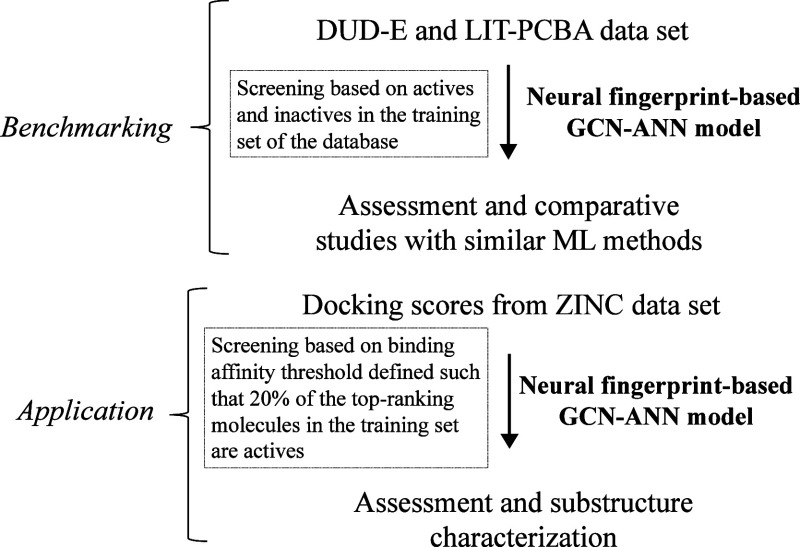


The overall study was compartmentalized into two phases: benchmarking and application (Scheme 1). In the first phase, the machine learning architecture, developed in this study (*vide infra*), was tested on two popular databases standardized for benchmarking molecular screening algorithms. The first one is the Database of Useful Decoys: Enhanced or, DUD-E,^44^ which is comprised of active ligands from ChemBL^45^ as well as synthetic, inactive decoy molecules. The second data set, LIT-PCBA^46^ is a larger, unbiased data set consisting of active and inactive ligands based on PubChem^47^ bioassay data. A total of 12 targets were chosen, based on their diversity of chemical and biological functions as described later. The screening was done using actives and inactives in the training pool abstracted from the database. In the second phase, the model was applied to six target proteins, selected based on their diverse biological functions and relevance to diseases (Scheme 1). To preserve the diversity of the chemical space and avoid user biases, a set of molecules randomly sampled from the ZINC database^48−50^ was used for this study. After preprocessing and docking (see below), a total of 49,539 molecules was retained. The actives were determined by a binding affinity threshold that separates the data set such that the top 20% of molecules based on docking score (i.e., topmost tightly bound) are classified as hits.

### Preparation of Molecular Library and Targets

ZINC database stores the structural information on a large number (>1 million) of small molecules as SMILES^51−53^ or “simplified molecular-input line-entry system”. In SMILES format, the information on a small molecule is reduced to a single-line ASCII string of characters.^52^ This contains atoms in English letters, while bonding and stereochemistry are expressed by using special characters. Using a set of home-built scripts that utilize Open Babel commands, each of these strings was converted to three-dimensional structural forms. Finally, AutoDockFR^40^ was used to store them in a docking-ready protein data bank with partial charges or PDBQ format. In parallel, each target protein structure was downloaded from protein databank.^37^ The receptor and ligand molecules were separated using VMD. Hydrogens were added to both receptor and ligand molecules using AutoDockFR^40^ before storing them in PDBQ format.

### Molecular Docking

High-throughput docking was accomplished by shell scripts that can parallelize a number of jobs using the GNU-parallel tool^54^ across multiple nodes. The binding affinity is usually expressed quantitatively by the binding enthalpy, which is estimated via a scoring function to account for interactions primarily involving hydrogen bonding, van der Waals, desolvation, and electrostatic energies (eq 1):
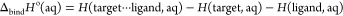
1where *H*(target···ligand, aq), *H*(target, aq), and *H*(ligand, aq) represent the aqueous enthalpy of the ligand-bound target, the unbound target, and the unbound ligand, respectively. The binding affinities (Δ_bind_*H*°(aq)), obtained from the docking output, were randomly separated into three train-validation-test groups with 70, 15, and 15 percentage splits, respectively. The greater binding affinity corresponds to more negative Δ_bind_*H*°(aq) values. A threshold of Δ_bind_*H*°(aq) was calculated based on the statistical representation of the training set data for a specific target active site (*vide infra*).

### Core Architecture

**Scheme 2:**
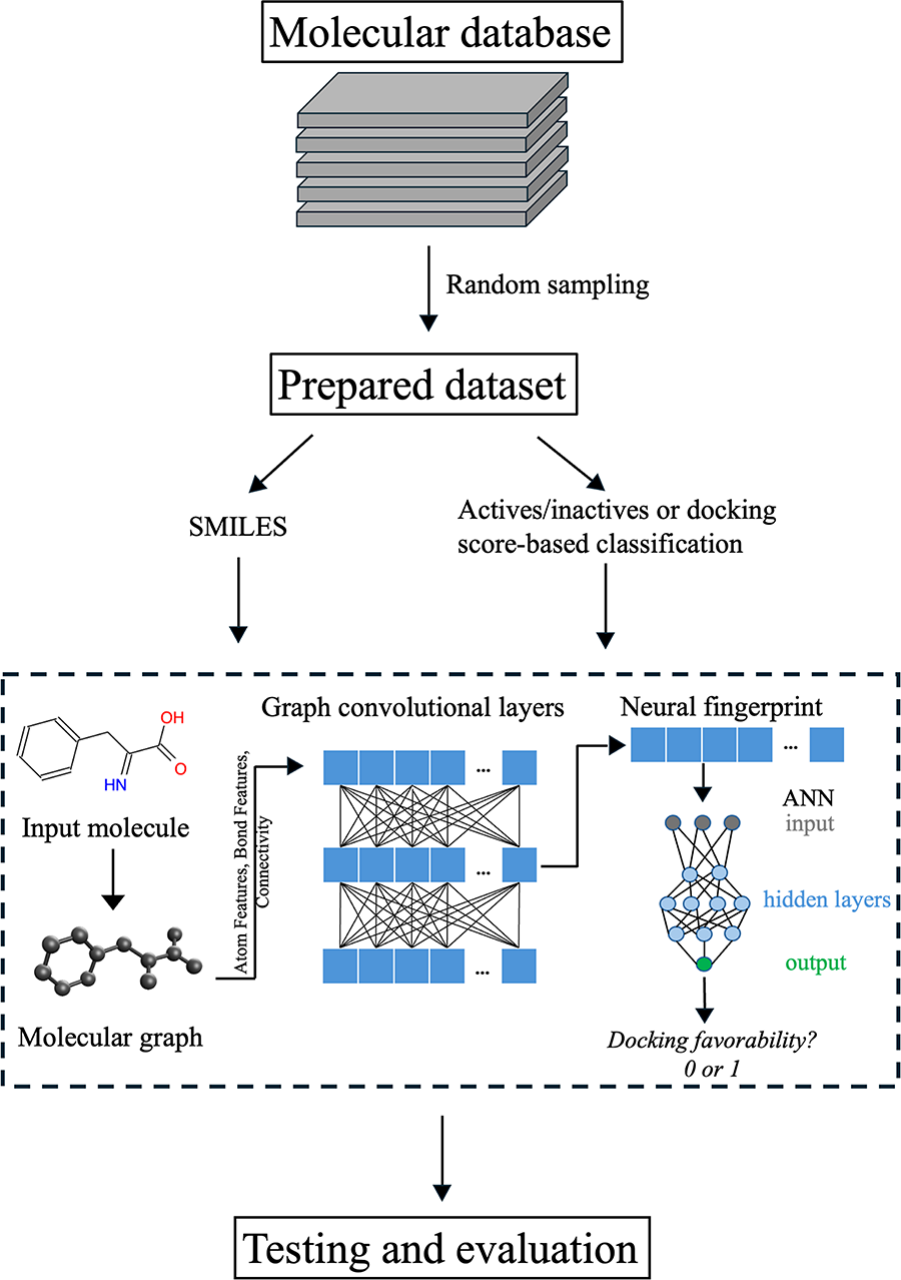


The core architecture of the graph convolutional network augmented with an artificial neural network (GCN-ANN) model is described below. This consists of fingerprint generation via a graph convolutional neural network which is then mapped to an artificial neural network (ANN) (Scheme 2).

#### Graph-Based Convolutional Neural Fingerprint

A molecular graph-based deep learning algorithm has been used to encode molecular substructure and generate fingerprints. The graph convolutional neural network (GCN) fingerprints were generated by using the method outlined in the paper of Duvenaud et al.^55^ This method is a significant modification of Morgan’s algorithm,^56^ which generates extended connectivity fingerprints (ECFP).^57^ Similar to the case of ECFP generations, each molecule is represented as a graph, with atoms as vertices and bonds as edges, however, instead of a hash function to aggregate the information, a group of learnable weight matrices is used in GCN resulting in a task-based encoding of the molecule.^58^ Thus, instead of a single ECFP input in each iteration, the algorithm involves graph-based convolutions to carry out weight and bias optimizations within the neural network architecture (Scheme 2).

The molecular graph is defined using three matrices, a matrix representing specific features of each atom in the molecule, a matrix representing specific features of each bond in the molecule, and a matrix representing the connectivity of atoms and bonds in the form of a graph (Scheme 2). The atom feature matrix contains information about the atoms that make up the given molecule alongside the degree of the atom (number of atoms covalently connected), the implicit valence, the bonded number of hydrogens, and aromaticity. Furthermore, the bond matrix denotes whether each bond is a single, double, or triple bond and whether they are involved in aromatic, conjugated, or constitute a ring structure. These features are all one-hot encoded; i.e. the categorical information is converted to binary format such that each category in the original variables is stored in binary columns as 0s and 1s. The last matrix is very similar to a simple connectivity matrix, with extra information characterizing the unique bond, which connects any two atoms. These matrices represent the input into the overall machine learning structure, specifically the convolutional model which generates the neural fingerprint, an optimized, trainable analog to general chemical fingerprints like the ECFP.

In the convolutional model, at each layer, a one-dimensional fingerprint of a specified length was created, which is a real-valued vector. The process of creating these fingerprints was similar to the generation of ECFP. However, differentiable functions replaced nondifferentiable functions in the generative algorithm, in turn allowing the inclusion of trainable weights and biases in this process, which were optimized during the training process. Some examples include the nondifferentiable hash and array-indexing functions that can be replaced by the continuous and differentiable sigmoid and softmax functions. The fingerprints at each convolutional layer are connected just like an artificial neural network (ANN). Typically, such a model is still called convolutional, as the generation of the fingerprints involves the combination of information from the three matrices consisting of atom features, bond features, and the molecular graph. These three layers of information are embedded into these fingerprints in a usable manner. The last layer produces the final fingerprint which is then mapped to the ANN (Scheme 2). Note that the trainable weights and biases in the convolutional fingerprint generation process were not trained separately to the ANN trainable parameters. The backpropagation of the loss from the binary ANN output included the trainable weights and parameters used in the generation of the fingerprint. Hence, this strategy enabled the fingerprints from molecules to be optimized based on the task presented to the model, which in the present scenario is predicting actives of a certain protein target.

#### ANN-Based Binary Classification

This ANN is a binary classification model based on a calculated binding affinity threshold. The Adam optimizer^55^ was used to optimize the parameters of the neural network during training. In addition, a hyperparameter search was carried out through a simple grid search technique with unique combinations of the following hyperparameters: weight decay, learning rate, dropout frequency, batch size, and fingerprint length (Table 1).

**Table 1:** Set of Hyperparameters Used in the Study to Train Models for Each Protein^a^

Hyperparameters	Values
Learning Rate	0.0001, 0.0003, 0.001
Weight Decay	0, 0.0001
Batch Size	128, 256
Dropout	0

a In total, 18 unique hyperparameter sets are trained on. Fingerprint length and hidden features are constant at 32 and 64, respectively.

Custom initialization was used in regard to both the ANN and fingerprint weights and biases. Fingerprint weights were initialized based on a normal distribution of the Xavier initialization method,^17^ which was shown to be favorable for the sigmoid activation function. Bias was initialized to a constant 0.01 for both the ANN and fingerprint. At each convolutional layer (including the initial input layer), an output layer transforms the associated atom features into a real-valued vector: the fingerprint. The vector sum of these intermediate fingerprints resulted in the final fingerprint, which was mapped to the first layer of a simple ANN.

Compared to existing works, the architecture presented has three amalgamated features that result in a novel architecture: the foundational work by Duvenaud et al.^55^ to build up a neural network architecture for fingerprint generation, a binary classification-based ANN to predict favorability in terms of binding affinities between a certain molecule and protein, and the connection of both of these separate architectures into one, hence optimizing both the fingerprint-generating and ANN architectures together. As a result, it is shown that a small sample of <50,000 molecules is enough for the architecture presented to capture the entire chemical space in relation to efficient binding affinity prediction.

The above architecture has some similarities to the reported study of Menke et al.,^59,60^ in particular to the initial use of the convolutional neural network for producing the fingerprints, but differs considerably in the final phase, where the fingerprints are extracted from the fully connected convolution layers. Instead, our algorithm follows the study of Duvenaud et al.,^50^ and the neural fingerprint is generated from the optimization of the weight matrices. This fingerprint is then fed to an ANN for binary classification, effectively stitching together the graph-based GCN and ANN that output and input the neural fingerprint, respectively. Hence, our architecture (abbreviated hereafter as GCN-ANN) not only includes the fingerprint generation, but also classification, and the fingerprint is optimized based on the loss calculated from the binary classification, described above.

### Techniques to Induce a Balanced Data Set

In a statistically unbiased sample, binary classes comprise 50% of the data set, enabling the model to be trained on wider features that define each class. However, this is not true for most virtual screening applications, benchmarks, and data sets. Usually, the subset of active ligands for a receptor is very small in relation to the certain chemical space or data set being studied. Such imbalances must be addressed to ensure that a machine learning model’s predictive capabilities apply to both the majority and minority classes of data. Thus, for training, class weights were calculated based on the ratio of virtual hits/active ligands and inactive molecules. These class weights act as numerical multipliers to the loss function, thereby penalizing the misclassification of the minority class (in our case, active ligands) to a greater extent than the majority class. This is done in hopes of pushing the model toward an explainable grasp of the substructures that are common in active ligands rather than solely predicting all molecules as nonhits. Oversampling was also tested, both independently and in tandem with the class weights approach, however, it seemed to not effectively solve the imbalance issue due to the large amount of copying of the same active ligands.

### Benchmarking with Standard Databases

As outlined before, benchmark studies were carried out to test the performance and feasibility of the machine learning model using two data sets (DUD-E^44^ and LIT-PCBA^46^) comprising of experimentally determined actives and inactives as well as computationally generated decoys. To keep a balance between the resources and the challenges of the study, we limited our benchmarking studies to a total of 12 drug design targets.

### Evaluation Metrics

All metrics presented were solely used to quantify results for the neural network architecture’s final predictive capabilities on the test data set. Precision is a general metric that quantifies the proportion of hits predicted by the neural network that were true hits based on their Δ_bind_*H°*(aq) values as in eq 2
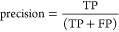
2where true positives (TP) refer to the number of predicted hits that were true hits and false positives (FP) refer to the number of nonhits that were predicted as hits. Recall is another metric that goes hand-in-hand with precision and relays the proportion of actual hits that were predicted as hits by the neural network (eq 3)
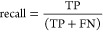
3where false negative (FN) refers to the hits that were predicted as nonhits by the neural network. The receiver operating characteristic (ROC) and area under the curve (AUC) is a metric often used in binary classification problems. The ROC curve plots the true positive rate (tpr) against the false positive rate (fpr) at different classification thresholds. The AUC, ranging from 0 to 1, is then called the ROC AUC. A higher AUC generally indicates better performance at binary classification.

The area under the precision-recall curve or PR-AUC plots the precision and recall at different probability thresholds to visualize the trade-off between the two at a given probability threshold. The area ranges from 0 to 1 and, generally, higher values indicate better, more consistent precision and recall for a model.

The predictive enrichment probability (PEP) is expressed as the probability of the model predicting a molecule as a hit given that the molecule is really a hit. In particular, this probability ranges over the Δ_bind_*H*°(aq) values that are lower than the threshold defined prior to training the model. In theory, this metric indicates how well a model can capture binary hits over a continuous distribution of values.
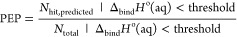
4where *N*_hit,predicted_ is the number of molecules predicted to be hits and *N*_total_ is the total number of molecules.

The F_1_ score is defined by the harmonic mean of the precision and recall as in eq 5:
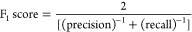
5

Enrichment within a set of *N*-ranked molecules, defined as the Top *N* enrichment, was calculated using eq 6:
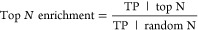
6where TP | top N is the number of TP found within the top N-ranked molecules by the algorithm, and TP | random N as the number of TP found within N randomly sampled molecules. The enrichment factor (EF) within a set of N-ranked molecules was computed by using eq 7:
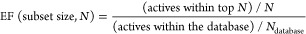
7where *N*_database_ is the number of molecules in the database.

## Results and Discussion

The GCN-ANN model described above was benchmarked using standardized databases before extending the study to several drug targets. These benchmarking experiments were done to validate the model’s performance on data sets with active ligands. On the other hand, for the six drug targets, the model was used to classify ligands based on computationally obtained docking scores.

### Benchmarking with DUD-E Database

From DUD-E, five different protein systems were chosen. The rationale for choosing these targets is the availability of sufficient experimental data, the variability of their substrates, and the diversity of their biological functions. Involved in key cellular signaling processes, these five protein systems are adenosine A2a receptor (AA2AR),^61^ cyclin-dependent kinase 2 (CDK2),^62^ estrogen receptor alpha (ESR1),^63^ tyrosine-protein kinase (SRC),^64^ and vascular endothelial growth factor receptor 2 (VGFR2).^65^ These targets were used to investigate the efficacy of the proposed architecture with experimentally determined active ligands. Metrics for analyzing performance include ROC and PR curves, and EF. The latter is a metric that quantifies the ratio of true active ligands in a subset of predicted actives and true active ligands in the overall data set. Therefore, it offers a measure of the quality of the predicted data set. Table 2 lists the number of active ligands and synthetic decoys provided by DUD-E for the five protein systems investigated.

**Table 2:** Counts of Active Ligands and Decoys for the Five Protein Systems Used for Benchmarking

Ligand type	AA2AR	CDK2	ESR1	SRC	VGFR2
Active ligands	482	474	383	524	409
Decoys	31,550	27,850	20,685	34,500	24,950

Being a widely utilized benchmark for docking programs, certain concerns about the biased generation of the DUD-E decoys data sets have been raised, including in the work of Chen et al.,^32^ which describes two relevant forms of bias: analogue and decoy bias. Analogue bias refers to the tendency of active ligands across targets of functional similarity to be correlated in chemical space. For models that input protein–ligand features, this may present a problem as patterns learned from the ligands of one protein may hinder the model from learning features of ligands of another protein and instead focus on mundane patterns in chemical space. For models that only input ligand features, this bias may result in continuing to identify ligands within the narrow chemical space with similar substructures, topology, etc. The second and more relevant class is decoy bias which is caused by similarities between decoy molecules generated through biased approaches by the researchers and creators of DUD-E.^32^

**Figure 1:**
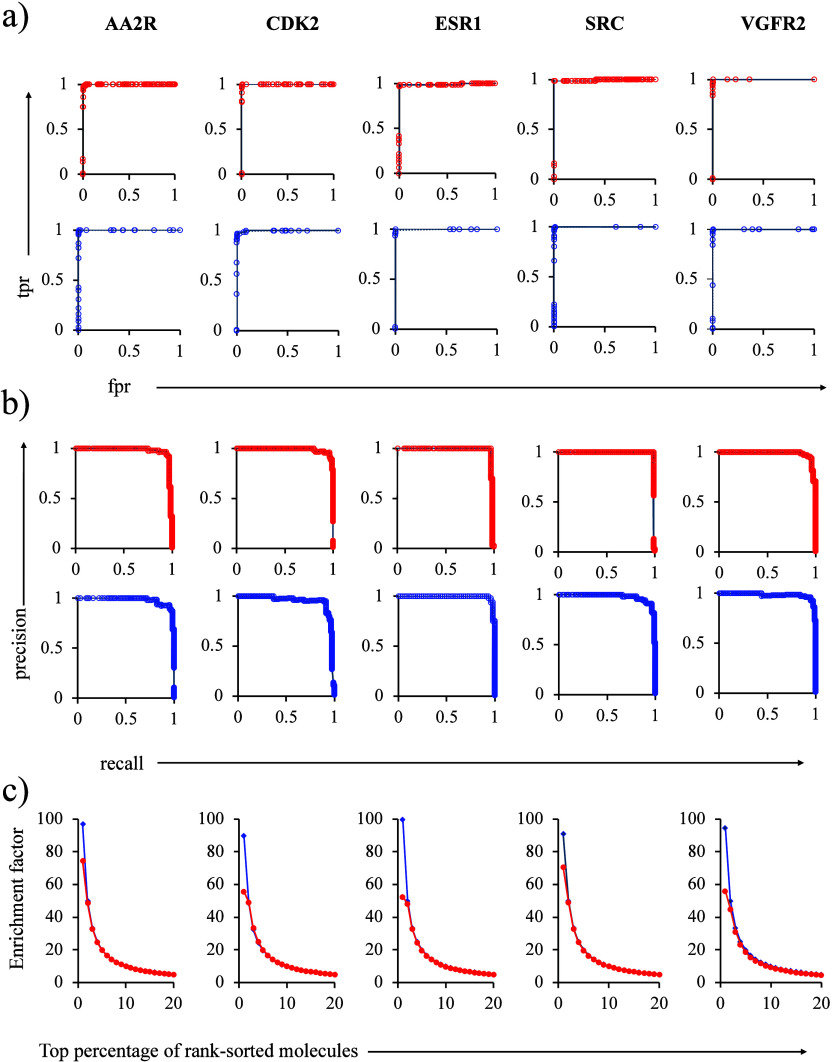
Assessment of the models using (a) ROC curves, (b) PR curves, and (c) EF plotted against top percentage of rank-sorted molecules for the five proteins listed in Table 2 for the DUD-E benchmarking data set composed of active ligands with decoys (in red) and actives with inactive molecules sampled from the ZINC database (in blue).

In order to address the inherent decoy bias in the data set, benchmarks for each protein system were undertaken twice: with a data set with the original active ligands and decoys and a data set with the original active ligands and 49,539 randomly sampled ligands from ZINC database. By sampling these molecules, the chemical space of the data set was effectively expanded, removing any patterns among decoys that may induce patterns between decoys. The performance metrics containing ROC curves, PR curves, and enrichment factors (EF) for each of the five protein systems are shown in Figure 1. These metrics revealed that the GCN-ANN model behaved like a perfect classifier for all five protein systems. The ROC curves and PR curves (Figure 1a,b) indicate that the model was able to learn near-perfect classification boundaries within the chemical space of the decoys and actives as well as the expanded chemical space of the ZINC ligands and actives.

Moreover, the enrichment factor (EF) of the proteins on test sets (eq 7) ranged from top 1% ranked subsets of the predicted data set to top 20% of the predicted data set (Figure 1c). The recognizable, exponential curve of the EF for all the proteins indicates a correlation between high ranks and more dense subsets of active ligands. The ratio between EF_max_ and EF is 1 for all proteins except VGFR2 which has a ratio of around 0.88 for data sets with decoys (data not shown). On the other hand, for data sets with randomly sampled ligands, the ratio between EF_max_ and EF is slightly less impressive with the range being 0.88–0.95 for four proteins but was a bit low (0.61) for ESR1. This may be due to the improbable lack of active ligands from the expected mean in the test data set for the ESR1 model. Nevertheless, all the metrics highlight the model’s efficacy in selecting hits within data sets comprising designed inactive decoys and randomly sampled inactive ligands.

Table 3 illustrates the ROC-AUC values ascertained from the GCN-ANN architecture of this study, and two other models: the central Morgan Fingerprint model developed by Gentile et al.^19^ and a Distance plus Attention for Affinity Prediction (DAAP) model by Rahman et al.^6^ that incorporates distance-based features in predicting protein–ligand binding affinity. These ROC-AUC values are for benchmarks with data sets including decoys. Note that Gentile et al.^19^ supplemented the DUD-E data set active ligands with randomly sampled ZINC ligands. Since we found insignificant differences in ROC curves between both benchmarked data sets, we have provided the decoy-based data set’s results. The ROC-AUC values for the proposed model are consistent across proteins and represent very significant increases in the performance of the model in the context of the binary classification problem.

**Table 3:** Comparisons of ROC-AUC Values between the Proposed Models and Two Other Proposed Models for Predicting Protein–Ligand Binding Affinity^a^

Protein targets/machine learning models	AA2AR	CDK2	ESR1	SRC	VGFR2
Morgan fingerprint^56^	0.52	–	0.91	–	0.62
DAAP^6^	0.68	0.75	–	0.63	–
GCN-ANN model (this study)	**0.97**	**0.97**	**0.96**	**0.99**	**0.98**

a Omitted cells represent values for which protein systems were not benchmarked. The best value for each protein is highlighted in bold.

### Benchmarking with LIT-PCBA Database

To substantiate the results of the DUD-E benchmark, benchmark studies were carried out with seven protein systems provided by the LIT-PCBA data set.^46^ A relatively contemporary benchmark, LIT-PCBA is specifically catered toward machine learning-based virtual screening methods for binding affinity prediction. By processing 149 dose–response PubChem bioassays^47^ using various techniques including asymmetric validation embedding (AVE),^66^ the data set is unbiased, and benchmark results between various ML models are standardized, valid, and reliable. The seven protein systems from the database were chosen based on a significant diversity of substrates and sufficiently large data sets of active and inactive ligands for training. These proteins are aldehyde dehydrogenase 1 (ALDH1),^67^ flap endonuclease 1 (FEN1),^68^ b-glucocerebrosidase (GBA),^69^ lysine acetyltransferase 2A (KAT2A),^70^ mitogen-activated protein kinase 1 (MAPK1),^71^ pyruvate kinase M2 (PKM2),^72^ and vitamin D3 receptor (VDR).^73^Table 4 displays the number of active ligands and inactive ligands contained in the data sets of each of the seven proteins.

**Table 4:** Number of Active Ligands and Inactive Ligands for Seven Protein Systems Provided by LIT-PCBA

Ligand type	ALDH1	FEN1	GBA	KAT2A	MAPK1	PKM2	VDR
Active ligands	7168	369	166	194	308	546	884
Inactive ligands	137,965	355,402	296,052	348,548	62,629	245,523	355,388

As evident from both benchmark studies, a significant difference exists between the data sets provided by DUD-E^44^ and LIT-PCBA^46^ in terms of the sizes (i.e., the number of molecules), thereby, resulting in the class imbalance demonstrated by low active to inactive ligands ratio (Tables 2 and 4).

The ROC-AUC values are used as metrics to analyze the performance of the model on the LIT-PCBA benchmark data sets. Table 5 compares the ROC-AUC values obtained by training and testing on the aforementioned seven protein systems, compared across three architectures, FP-GNN (Cai et al.^7^), GEM-2 (Liu et al.,^74^ and EGT + TGT-At-DP (Hussain et al.^75^). FP-GNN utilizes molecular fingerprints and a graph neural network (GNN) to predict binding affinity.^7^ GEM-2^74^ uses various tracks to transform features into representations to predict molecular properties including binding affinity. Lastly, EGT + TGT-At-DP^75^ is a combination of an Edge-Augmented Graph Transformer (EGT) and the Triplet Graph Transformer (TGT) alongside a distance predictor.

**Table 5:** Comparison of ROC-AUC Values of Binding Affinity Binary Classification Tasks on Seven Protein Systems from the LIT-PCBA Dataset Across Three Other Architectures^a^

Protein targets/machine learning models	ALDH1	FEN1	GBA	KAT2A	MAPK1	PKM2	VDR
FP-GNN^7^	0.77	0.89	0.75	0.63	0.77	0.73	0.77
GEM-2^74^	0.77	0.93	0.82	0.67	0.72	0.72	0.80
EGT+TGT-At-DP^75^	0.81	0.96	0.84	**0.75**	0.74	0.78	0.83
GCN-ANN model (this study)	**0.82**	**0.97**	**0.93**	0.73	**0.82**	**0.88**	**0.90**

a The best value for each protein is highlighted in bold.

Across all proteins, the ROC-AUC values computed by GCN-ANN model (Table 5), developed in the present study, matched with or exhibited significant improvement over the three aforementioned implementations as reported by Hussain et al.^75^ To remind the readers, these architectures vary significantly in terms of their complexity and mechanisms for molecular property/binding affinity prediction. As the ROC-AUC values relate the ability of the classifier to learn ligand (or protein–ligand) features in order to establish a decision boundary in chemical space, it can be concluded that the proposed model learns ligand features relevant to binding affinity.

Furthermore, the above comparative analysis demonstrates that the proposed GCN-ANN architecture not only learns on relatively smaller data sets but also performs consistently well for the larger LIT-PCBA training data set with significant class imbalances (i.e., low active-to-inactive ligands ratio). Therefore, this feature of the architecture presents inhibitor screening opportunities for smaller laboratories with limited resources as well as laboratories capable of scanning larger molecular data sets containing a significantly smaller ratio of active to inactive ligands.

### Evaluating the Screening Efficiency of the GCN-ANN Model on Six Targets

**Figure 2:**
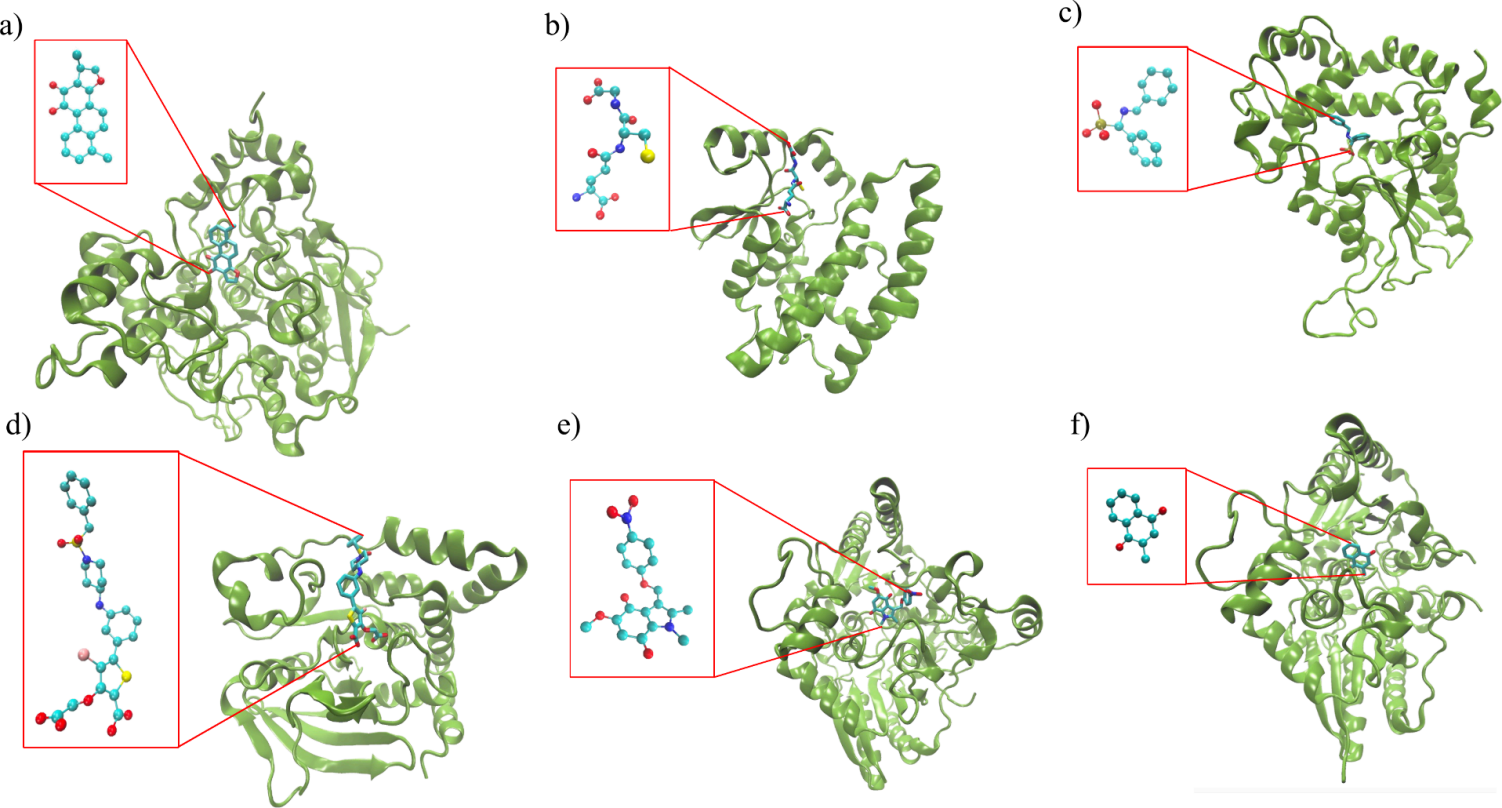
Folds of the enzymes and the active site-bound small molecules (shown in the insets) for (a) AChE with dihydrotanshinone I, (b) GST with glutathione, (c) PAP with a-benzyl-aminobenzyl-phosphonic acid, (d) PTP1B with ligand ID: 527, (e) NQO1 with ligand ID: ES936, and (f) NQO2 with menadione.

To further examine the screening efficacy of the GCN-ANN architecture, developed in the present study, six enzymes were chosen that are well regarded as potential therapeutic targets for various diseases. These enzymes were selected as they exhibit distinct enzymatic mechanisms and as shown in Figure 2, these enzymes have different folds, and the bound small molecules are diverse in terms of their molecular features. The following is a brief description of the structure–function of these enzymes related to their recognition as drug targets.

#### Acetylcholinesterase

Abbreviated as AChE, hereafter, is responsible for the regulation of neurotransmission through the degradation of acetylcholine (the neurotransmitter) in synapses of the nervous system.^76^ Inhibitors of this enzyme are sought as they can be used as therapeutics for the treatment of disease and protection against nerve agents. The X-ray crystal structure (Figure 2a) representing the inhibitor dihydrotanshinone I-bound target (PDB code 4m0e(76)) was used for this study.

#### Glutathione S-Transferase

Abbreviated hereafter as GST^77^ is responsible for adding electrophilic group to glutathione (the tripeptide formed with cysteine, glycine, and glutamic acid) and is responsible for detoxification. Additionally, they are involved in promoting tumor pathogenicity and chemoresistance.^78^ The current study was based on the protein structure (PDB code: 1pkw(77)) in complex with glutathione (Figure 2b).

#### Prostatic Acid Phosphatase

The prostatic acid phosphatase enzyme (abbreviated hereafter as PAP) is responsible for the malignant growth of cells.^79^ Inhibitors of this enzyme can be used as therapeutics for prostate cancer. The enzyme (PDB code: 1nd5(79)) falls into the subclass of protein tyrosine phosphatase and is responsible for the dephosphorylation of epidermal growth factor receptor. The a-benzyl-aminobenzyl-phosphonic acid-bound structure is shown in Figure 2c.

#### Protein Tyrosine Phosphatase 1b

Also belonging to the class of protein tyrosine phosphatase, this enzyme (abbreviated as PTP1B) regulates negatively insulin.^80^ This enzyme is an attractive target for type 2 diabetes and obesity and the drug (5-(3-{[1-(benzylsulfonyl) piperidin-4-yl]amino}phenyl)- 4-bromo-3-(carboxymethoxy) thiophene-2-carboxylic acid; ligand id: 527)-bound X-ray crystal structure (Figure 2d) used in the study belongs to the PDB code: 2qbp.^80^

#### NAD(P)H:Quinone Oxidoreductase Type 1

This oxidoreductase enzyme is responsible for protecting cells against cellular toxicity due to free radicals.^81^ This enzyme (abbreviated as NQO1) is overproduced in cancerous cells and therefore selective inhibitors of the enzyme have strong chemotherapeutic potential. The present study is carried out on the 5-methoxy-1,2-dimethyl-3-(4-nitrophenoxymethyl)indole-4,7-dione (ligand ID: ES936)-bound X-ray crystal structure (Figure 2e) of the enzyme bearing the PDB code: 1kbq.^81^

#### NRH:Quinone Oxidoreductase Type 2

This is another oxidoreductase enzyme (abbreviated as NQO2) involved in regulating cellular toxicity and oxidative stress.^82^ The enzyme is targeted for anti-Alzheimer disease drug development. As illustrated in Figure 2f, the X-ray crystal structure of the menadione-bound enzyme (PDB code: 2qr2) was used in the study.^82^

### Parameters and Metrics for Benchmarking

Based on the essence of compound screening, the present model was optimized to retain molecules with relatively higher binding affinities while “filtering” as many molecules with relatively lower binding affinities. Therefore, the use of larger, preliminary data sets of small molecules would become feasible.

Using various metrics to benchmark, the performance of models was compared, both between the same architecture and between different architectures, e.g. convolutional fingerprint and Morgan fingerprint models. For a general metric, AUC measured the quality of the models’ predictions across different classification thresholds. To demonstrate how well these “filtration” architecture performs, increased significance was placed on the true positive rate (sensitivity). The purpose of compound screening is to retain the ligands that most favorably bind to a receptor. In turn, it is increasingly important that the number of false negatives was minimized in order to not discard any potentially promising compounds. Hence, sensitivity is maximized as much as possible without handicapping the model’s ability to be precise in its predictions.

All parameters are calculated based on final predictions using the test data set after training and validation. To simulate the imbalance between active and inactive molecules, the top 20% of molecules based on docking scores are set as hits across all benchmarks on the six proteins mentioned above. As a result, the training data set contained about 27,742 nonhits (inactives) and 6935 hits (actives). It is important to note that the hits may not necessarily be bioactive, but they are considered active/hits by the model in order to retain them after screening while filtering out the nonhits. The classification threshold is set to return a classification boundary with a recall value of 0.90 across all targets. This is done to ensure a consistent comparison among different proteins and other models. The pertinent parameters of all models using the neural network architecture are presented in Table 6.

**Table 6:** Recall, Precision, ROC-AUC, PR-AUC, and F1 Scores for the Six Protein Systems Studied^a^

target enzyme	Recall	Precision	ROC-AUC	PR-AUC	F1 score
AChE	0.90 (0.70)	0.57 (0.58)	0.94 (0.90)	0.80	0.70
GST	0.90 (0.70)	0.53 (0.59)	0.94 (0.90)	0.79	0.67
PAP	0.90 (0.71)	0.44 (0.34)	0.90 (0.86)	0.71	0.59
PTP1B	0.90 (0.82)	0.72 (0.72)	0.97 (0.95)	0.89	0.80
NQO1	0.90 (0.72)	0.56 (0.62)	0.94 (0.91)	0.79	0.69
NQO2	0.90 (0.71)	0.61 (0.59)	0.95 (0.90)	0.82	0.73

a All values are calculated based on model predictions on the test dataset after training. Values in parentheses represent values obtained by the Morgan fingerprint method proposed by Gentile et al.^19^ All values were calculated using the scikit-learn library and based on model predictions on randomly sampled test data sets. The values were obtained from the best-trained model. The uncertainties are within 0.02.

In Table 6, metrics are calculated based on a binary threshold which sets recall at 0.90 to provide a baseline for comparison among the protein systems. The precision and recall values (Table 6) seem to characterize both models appropriately. The Morgan fingerprint-based “deep docking” architecture and the proposed architecture seem to have comparable precision values across the six chosen protein systems. However, the advantage of the proposed GCN-ANN model can be observed by comparing the recall values, where improvements range from 0.08 to 0.20. This performance can be further quantified through the improvements in ROC-AUC which characterize performance on the binary classification problem. Although the presented F1 scores relay the increased significance placed on the recall of virtual hits, relatively high PR-AUC values for all proteins emphasize the performance of the proposed model regardless of the imbalanced classes (80% nonhits, 20% virtual hits). Moreover, the “deep docking” algorithm used an iterative docking protocol, but the present comparative analysis is carried out using only a single iteration of this protocol. The former study revealed that the threshold binding affinity cutoff remained quite unchanged until iteration 11. In other words, the docking algorithm was unable to filter out the best molecules, until it reached iteration number 12 or beyond. In contrast, the statistical evidence of the model generated in this study demonstrated that docking cutoffs of the predicted virtual data set adhered to the docking threshold set before training, even in the first iteration. As elaborated by the performance metrics in Table 6, the present study establishes that higher retention is being achieved even in the first iteration, giving a huge performance boost to the filtering process. Even using a smaller data set, the algorithm was able to map the chemical space in relation to substructure recognition for predicting binding affinity with great efficacy.

These fundamental binary classification parameters relay two important things: the nature of the imbalanced data set as well as the suitability of the model based on the protein being investigated. Both the ROC-AUC and PR-AUC values vary based on the protein. As a result, it is evident that the neural network architecture was able to capture the significant affinity (i.e., large negative values) for molecular substructures at a higher level for proteins such as PTP1B compared to PAP. Moreover, compared to other deep-learning-based studies,^18,19^ the ROC-AUC values seem to be more consistent across the wide array of proteins selected for this study. Although specific metrics may vary, this observation seems to substantiate the classifier’s ability to perform well at different thresholds for many different proteins.

#### Precision at Various Recall Thresholds

**Figure 3:**
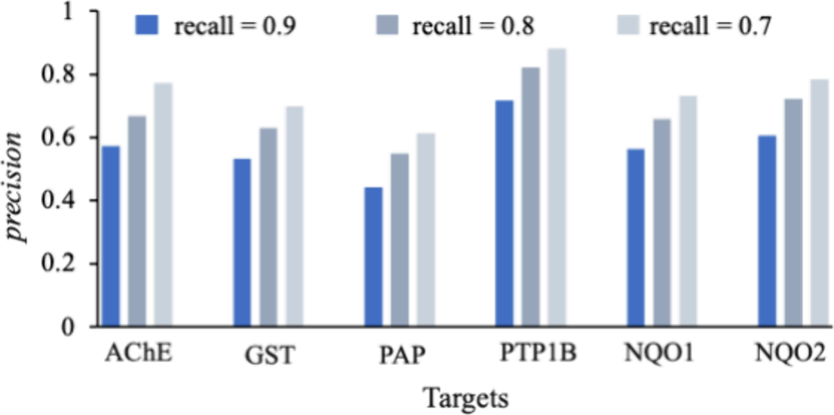
Variation of precision obtained with recall values of 0.7, 0.8, and 0.9.

Precision values (Figure 3) at various thresholds (0.7, 0.8, and 0.9) of recall demonstrate the gain of the model in terms of its performance. A comparison with the Morgan fingerprint method by Gentile et al.^19^ further highlights the performance gains by the GCN-ANN model. This illustrates that various thresholds can be set based on the needs of users and a precision-recall balance can be achieved for prediction. Regardless, the model presents expected trade-offs for said metrics, substantiated by Figure 3 as well as the PR-AUC values (*vide infra*).

#### Receiver Operative Characteristic and Precision-Recall Curves

**Figure 4:**
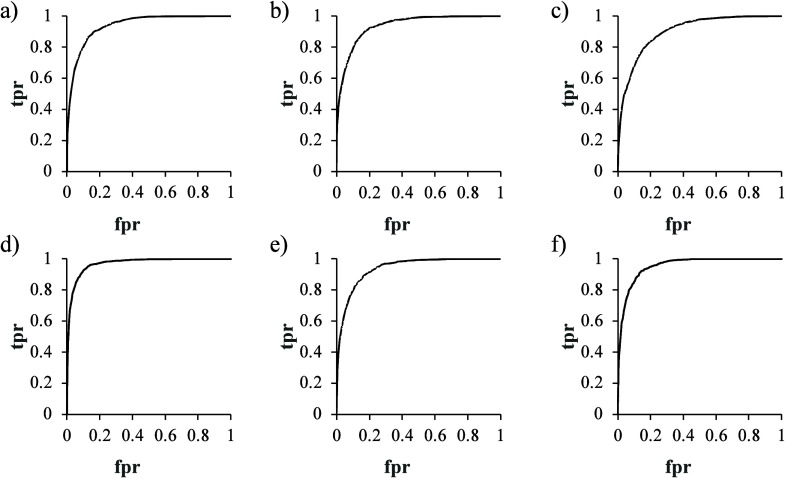
Receiver operative characteristic (ROC) curves for the six models, namely, (a) AChE, (b) GST, (c) PAP, (d) PTP1B, (e) NQO1, and (f) NQO2. The ROC-AUC values are given in Table 6.

The results consisting of receiver operative characteristic (ROC) curves, precision-recall (PR) curve, are presented (Table 6). These results are based on a hyperparameter grid search undertaken only a single time to ensure a notion of randomness in the quantitative performance across all proteins. Results described below are for single models chosen from the hyperparameter grid search. Parameters considered when choosing this “best model” include precision, recall, and average Δ_bind_*H°*(aq) of the predicted hits. The ROC curves of the six models (Figure 4), with respective AUC values in Table 6.

**Figure 5:**
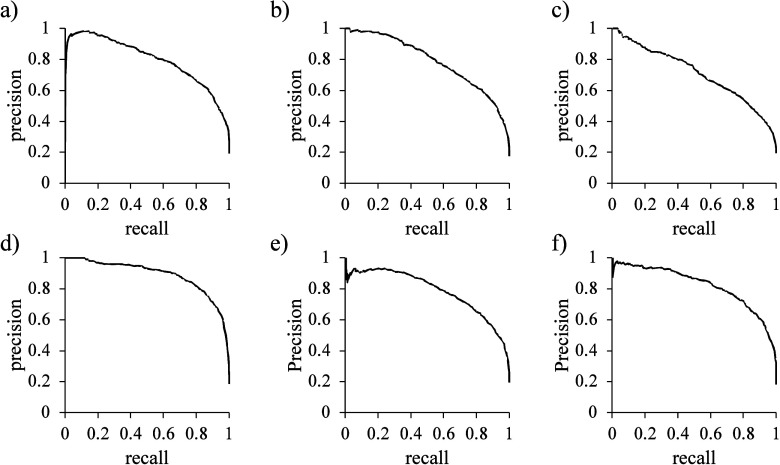
Precision-recall (PR) curves for the six models, namely, (a) AChE, (b) GST, (c) PAP, (d) PTP1B, (e) NQO1, and (f) NQO2. These curves are plotted based on the best selected model for each protein alongside corresponding PR-AUC values given in Table 6.

Moreover, the precision-recall curves (Figure 5) with corresponding AUC values have also been included in order to gauge the trade-off between precision and recall along various thresholds. However, in order to efficiently “filter” out molecules with higher negative Δ_bind_*H°*(aq) values, it is important for these models to be able to also classify the said molecules effectively.

#### Mean Predicted Binding Affinity and Binding Affinity Threshold

**Figure 6:**
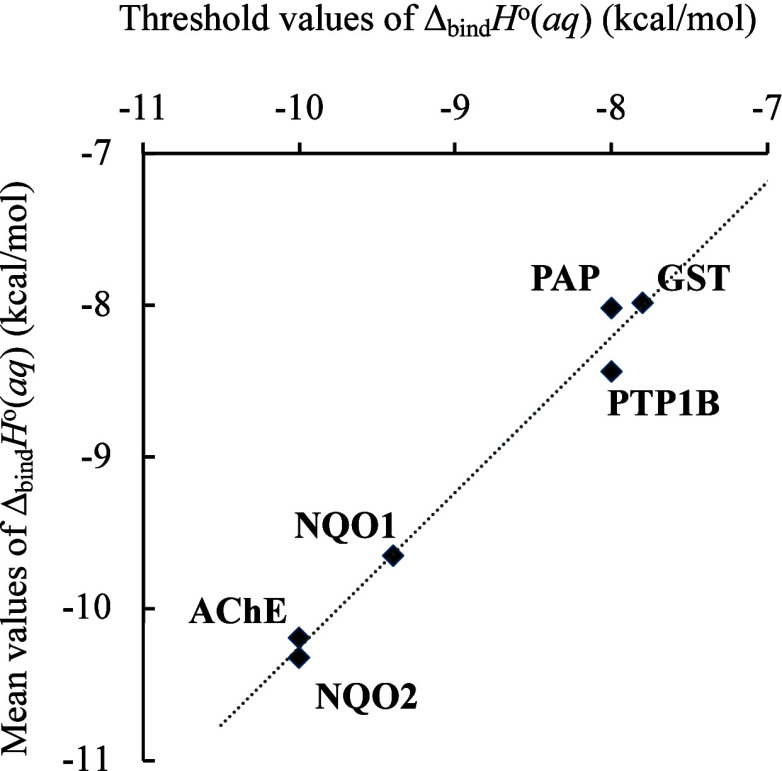
Mean binding affinity (value of the data set for virtual hits compared to threshold scores for ligands across the six tested proteins).

The model’s ability to virtually screen can be furthermore quantified by the mean docking score (i.e., binding affinity) of the predicted virtual hits after classification and the original data set of molecules (Figure 6). For all proteins, an observed negative shift was noted for the mean predicted binding affinity across the data sets, when a specific binding affinity threshold was used, illustrating effective filtration of the ligands based not only on classification as hits and nonhits but also docking scores. These thresholds were calculated to split the data set into 20% virtual hits and 80% nonhits.

#### Predictive Enrichment Probability

**Figure 7:**
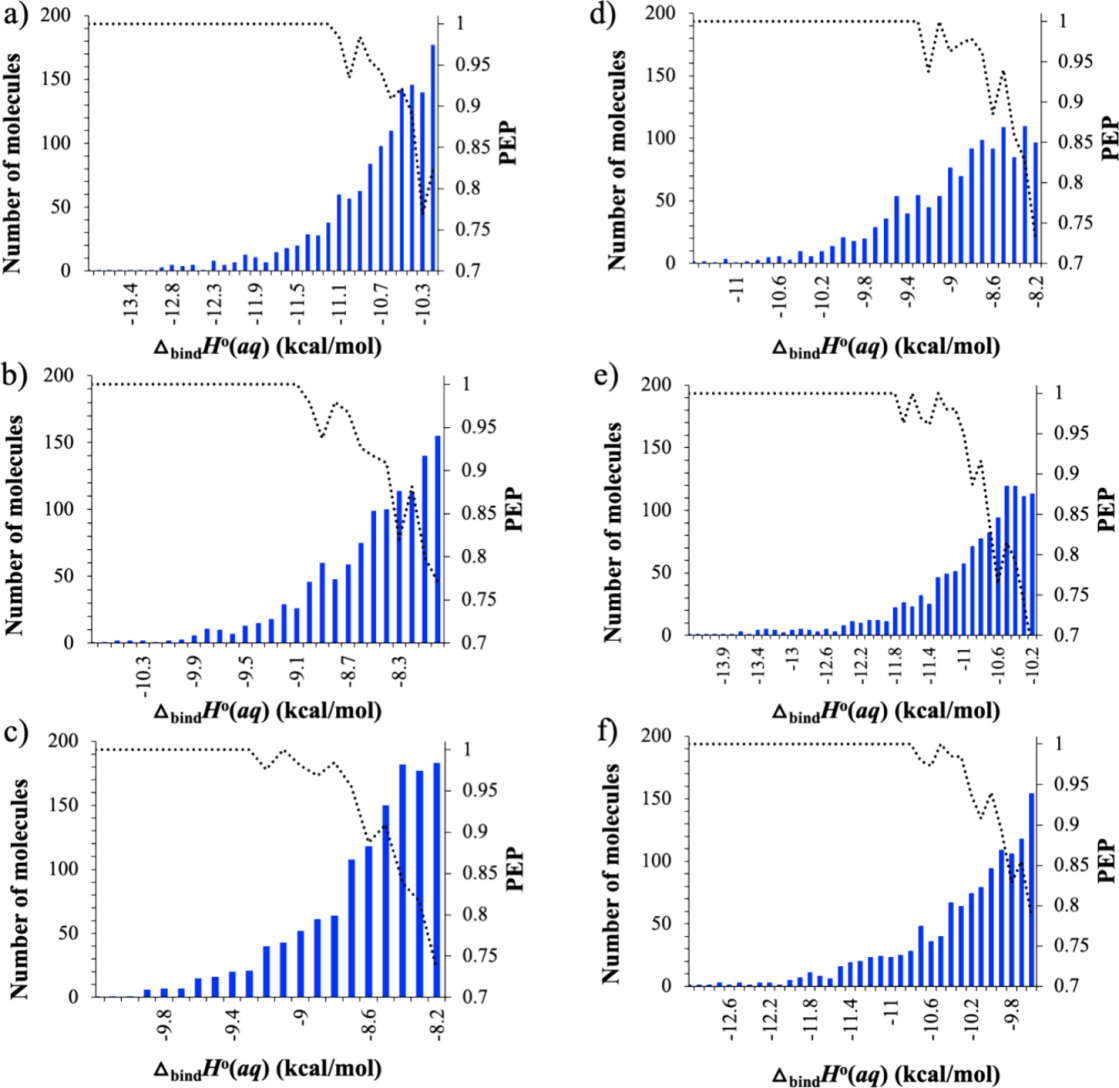
Predictive enrichment probability graphs and the total number of molecules with which the PEP was calculated over the range of Δ_bind_*H*°(aq) values in the binary “hit” range for (a) AChE, (b) GST, (c)PAP, (d) PTP1B, (e) NQO1, and (f) NQO2. Note that the counts displayed as bar graphs are the number of correctly predicted molecules (i.e., virtual hits) at each Δ_bind_*H*°(aq) value.

To further visualize and quantify the models’ abilities in correctly predicting those molecules that have a lower Δ_bind_*H°*(aq) value than the predefined threshold (e.g., recall), the predictive enrichment probabilities or PEP is calculated (eq 4) based on the set of hits in the test data set for each protein (Figure 7). The PEP was calculated at each binding affinity value lower (or equal) to the binding affinity threshold that was used in training. The granularity of the Δ_bind_*H*°(aq) values (eq 1) is found to be 0.01. The six graphs are shown in Figure 7 illustrating the PEP alongside the number of molecules in the test data set that are hits at each binding affinity value.

The PEPs for all proteins indicate that the neural network architecture can firmly grasp the molecular substructures that correlate to a lower binding affinity (Figure 7, toward the left of the graphs) and are almost always predicted correctly as hits by the neural network architecture. This is imperative in a good docking-based machine-learning model. It is necessary to filter out as many molecules while incurring the least amount of loss of tightly bound molecules to the protein, which in this case means the loss of molecules with a very low binding affinity. Without this characteristic, it would become increasingly infeasible to use such models in lieu of traditional docking techniques.

It is clear to see the effect of an imbalanced data set on the results based on the F_1_ score (eq 5) and ROC-AUC values (Table 6). Although recall is greatly maximized, precision remains relatively low. Although that is the purpose of the architecture, there may be further optimizations that can be implemented in regard to the architecture to enable the model to more performantly discard molecules with low binding affinity. Nevertheless, the extremely high recall attests to the architecture’s ability to retain molecules that favorably bind to the protein being investigated. Moreover, some proteins seem more favorable in investigating using this model than others (as was mentioned previously). In the case of PTP1B, around 93% of molecules in the bottom 20% of Δ_bind_*H*°(aq) values are retained whereas, based on the relatively high precision value, a good chunk of the overall data set seems to be reduced. All in all, the architecture quantitatively performs very well at retaining molecules meaningfully.

#### Top-N Enrichment

**Figure 8:**
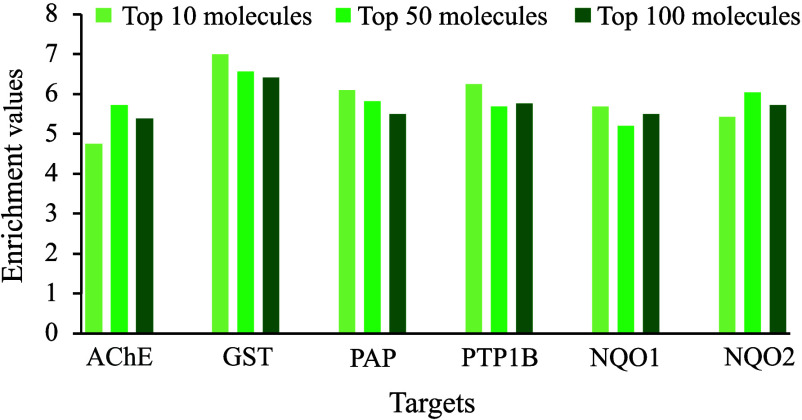
Top-N (*N* = 10, 50, 100) enrichment values for the targets.

Although the aforementioned metrics provide appropriate insights into the performance of the model as a learned function in solving a classification problem, further metrics are required to understand how well-enriched a predicted data set is. Top-N enrichment (eq 6) gives an estimate of the density of true positives among the top-N ranked molecules postprediction compared to that of the overall data set. Given that the threshold docking scores were set to only include the top 20% of ligands as virtual hits, a top-N enrichment (eq 6) value of 5 would be expected. In Figure 8, this expectation is substantiated, with most proteins overshooting the expected value of 5, indicating a higher level of enrichment in top-ranked molecules. Only one subset of the analysis, the top 10 molecules of AChE, did not reach this expected threshold. We attribute this to the inadequate chemical space conferred by the training data set; further studies with expanded data sets and, therefore, chemical space will be needed to address this slight inconsistency. As such, the model is not only classifying true positives at an above-expected rate, but its output logits demonstrate a correlation between rank and better binding ligands. This observation can be further corroborated by the predictive enrichment probabilities (Figure 8) in which the true positive rate for the ligands with the lowest binding affinity values are always retained.

#### Assessing Performance with CHEMBL Actives

Thus, the architecture developed in this study performed well in terms of screening efficiency using both the DUD-E and LIT-PCBA databases. Once the assessment of the screening was satisfactory, the model was applied to six targets using randomly sampled ZINC database molecules. For these targets, the docking score was computed and used to define hits and nonhits, and the architecture was successful in screening the top-ranking molecules from an unbiased pool of compounds. In addition, the performance of the model workflow was tested on the experimentally determined actives in CHEMBL data sets of for two proteins, AChE and PTP1B. Because of the adequate size, the assessment of the GCN-ANN architecture was done using a distribution of actives (<10,000 nM IC50) and inactives (Table S1). The results are fully consistent with the standards of evaluation metrics used in this study and validate the efficacy of the model based on both docking score and bioactive assay data. Taken together, the results (benchmarking and application) demonstrate that the model learns efficiently given a constrained chemical space and performs well with experimentally validated structures as well as docking results.

#### Efficiency of Training

**Figure 9:**
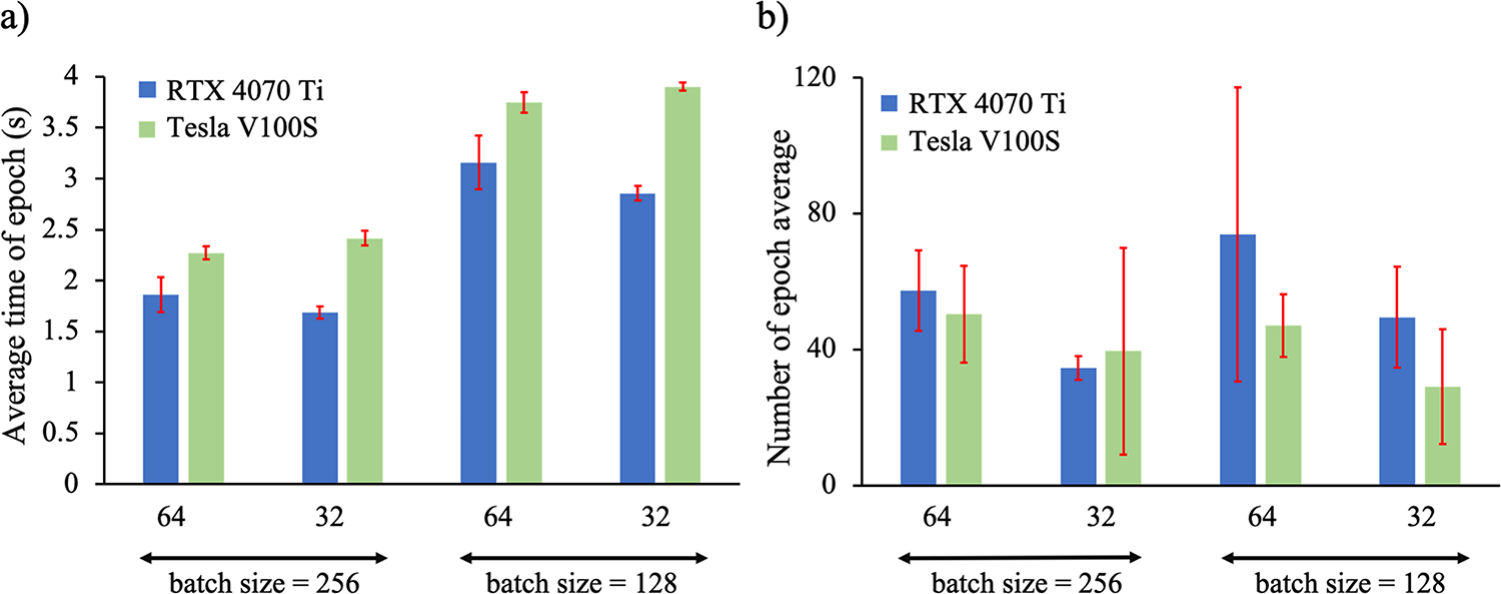
Quantitative gains obtained in various machines in terms of (a) the average time of the epochs and (b) the number of average epochs taken to train a model. In both cases, the gains differ based on the number of hidden features of the GCN-ANN architecture, represented by 32 and 64-bit architectures.

To compare the computing speed of the GCN-ANN architecture, two GPUs were used: an Nvidia RTX 4070 Ti which is commercially accessible and a Tesla V100S as part of the BOSE server at the University of Wisconsin Eau-Claire. Figure 8 quantifies both the average number of seconds taken to complete epochs during training (Figure 8a) and average number of epochs required to train (Figure 8 b). These values were calculated internally and across all six proteins. Furthermore, the data is split based on batch size utilized (128, 256), as well as the number of hidden features convolved across hidden layers of the fingerprint-generation network (32, 64). The total number of trainable parameters, 43,721 and 120,649, varied based on the hidden feature sizes of 32 and 64, respectively. Despite models with hidden feature sizes of 64 having about three times the number of parameters compared to models with hidden feature size of 32, the difference in time taken per epoch seems much smaller than a linearly scaled difference based on parameter size. This implies that both forward and backpropagation times seem to scale at a much lower rate relative to the overall time taken for a single epoch. As illustrated in Figure 9, larger models to accommodate larger data sets seem equally feasible. Readers might note that varying hyperparameters led to some variation in average epochs to train among the systems (Figure 9). Efficiency is of crucial significance for smaller laboratories and research groups, which may not have the time and resources compared to larger laboratories and organizations. Therefore, the comparative analysis is of significance in the sense that it demonstrates that not only the performance of the model is substantive, but also that the efficiency of the model is maintained across data sets of varying sizes.

#### Comparison of Substructures Using Fingerprint Activation Potentials

Fingerprint activation potentials were determined to identify the importance of substructures in a certain molecule with respect to its binding affinity toward a specific target. During the graph convolution process of fingerprint generation in the GCN, the activation potential of each atom in a molecule was calculated for each convolution layer and stored. The final layer’s activation potentials were then summed over to calculate the final fingerprint of a single molecule. Therefore, for a molecule the shape of the activation potential vector is the same as that of the final fingerprint vector with atomic contributions being ascertained from their respective numerical contributions to each fingerprint index. Across a larger test data set, the contributions of different atoms in different molecules can be sorted in order to retrieve the maximally activating atoms at specific fingerprint indices. These atom-specific potentials can then be mapped back to specific substructures (including said atoms) and their numerical contribution to the potential. Thus, it is possible to measure the significance of a substructure and its aggregate chemical information in the prediction of binding affinity.

**Figure 10:**
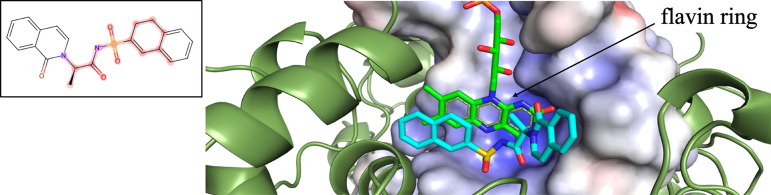
Visual assessment of the performance of the GCN-ANN model in predicting the substructure in potent inhibitors identified from their fingerprint activation values NQO1 from the docking pool used. The docked structure of the ligand into the active site is illustrated with surface, color-coded with electrostatic charges (positives in blue, neutral in white, and negatives in red).

Two such examples were chosen. The first example is an inhibitor of NQO1, which exhibited higher activation potentials involving a large part of the molecule. Visualization of the docked structure shows that the substructure identified by the GCN-ANN-computed fingerprint is indeed stacked on the flavin ring of the FAD cofactor of the enzyme (Figure 10). This indicates a stronger p–p interaction, which is known to be the significant contributor to the binding affinities of inhibitors of this group of flavoenzymes.^21^ The computed binding affinity of the inhibitor was −13.3 kcal/mol, which demonstrated a stronger binding, confirming that the hit was consistent to the geometry of the substructure identified by the GCN-ANN model (inset, Figure 10).

**Figure 11:**
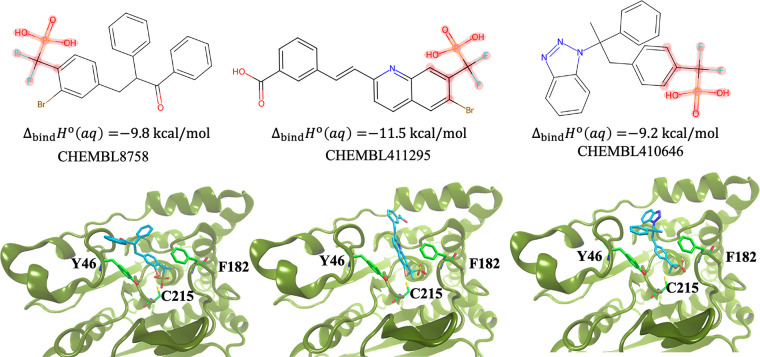
Visual assessment of the performance of the GCN-ANN model in predicting the substructure in potent inhibitors identified from their fingerprint activation values for three active molecules for PTP1B target obtained from CHEMBL. The highlighted section was identified from the fingerprint activation potential analysis from GCN-ANN and was verified by computational docking with the predicted affinities.

In the second case, three strong actives were chosen from the CHEMBL database (CHEMBL8758 (IC50 = 6 nM), CHEMBL411295 (IC50 = 7 nM), CHEMBL410646 (IC50 = 16 nM))^45^ of the PTP1B enzyme were chosen. Using the GCN-ANN model, the fingerprinting activation potential was generated and was mapped onto the molecules to generate two key substructures (Figure 11). As also highlighted in Figure 11, the docked structure of the molecule into PTP1B active site demonstrates that the phosphotyrosine mimicking phenyl difluoromethyl phosphonic acid part enters deep into the active site cleft reaching near the catalytic cysteine (C215) residue, which is known for its nucleophilic attack during dephosphorylation.^83,84^ As shown in Figure 10b, the hydrophobic side chains of (F182 and Y46) in the active site pocket (Figure 11) interact favorably with the inhibitor molecule. This substantiates the importance of the substructure identified by the GCN-ANN protocol.

Finally, permutation feature importance^85^ analyses (Figure S1) were done on the six best models for the six targets in the application phase of this work. Permutation feature importance, in this case, aims to quantitatively measure the contributions of each atom feature in the predictions of a trained model. This is done by randomly shuffling (permuting) a specific atom feature vector and measuring the change in accuracy between the original features and the permuted features. The analyses (Figure S1) demonstrate that the decrease in accuracy differs across targets, implying that the preference of atom features in the bound ligands is different among targets and hence is based on the characteristics of the protein active site.

## Conclusions

The perpetual increase of drug design-relevant candidates in easily accessible databases has certainly enriched prospects of identifying drug molecules that can selectively inhibit a specific target. Still, though, work in optimizing workflows and expediting methods for compound screening has not been able to keep up with this increase in indexed small molecules. Thus, using convolutional neural network-derived fingerprints a machine learning-based drug screening technique has been generated for faster compound screening. The architecture takes in, as input, various vectors that describe the structure of a molecule in terms of a graph. A differentiable, graph convolution-based fingerprint was calculated, and mapped to an ANN which then outputs a continuous value and is interpreted based on a binary classification problem. The performance accuracy and efficiency of this GCN-ANN was benchmarked using standard virtual screening databases, namely, DUD-E^44^ and LIT-PCBA.^46^ In almost all protein–ligand data sets available through the two aforementioned benchmarks, the proposed architecture represents a significant improvement in performance compared to contemporary, binding affinity-based machine learning solutions. Moreover, the varying sizes of data sets in the benchmarks, notably in LIT-PCBA, highlight the GCN-ANN model’s ability to learn chemical spaces effectively without bloating the architecture with unneeded parameters. This efficacy alongside the quantitative efficiency of the algorithm substantiates the feasibility of applying this architecture on data sets of widely varying sizes and chemical spaces. Furthermore, the analysis of the fingerprint-generating GCN part of the architecture highlights its capabilities of learning about ligand features that are relevant to the binding affinity of a target. This offers an appreciable amount of explainability to the model’s performance and predictions which can then be used for further pharmacophore research and drug design.

It was shown that the model performs well at retaining molecules that are classified as favorably binding to a specific protein. Based on the observed recall, ROC-AUC, and PR-AUC, the model performs reasonably well over a range of drug-targeted proteins. This includes data sets for ligands with computationally calculated docking scores and established benchmarks that include ligands with experimentally ascertained binding affinities. Compared to previous works, the ability of this architecture to maximally retain favorable molecules is much more significant without jeopardizing precision. However, various trained models present various characteristics, with some maximizing recall, maximizing precision, or finding a balance between both parameters. Hence, users of this model are enabled to choose models generated through the hyperparameter search based on use or circumstance. This demonstrates the plasticity of the neural fingerprint-based screening architecture. Furthermore, the relatively small working set of 50,000 molecules utilized as the overall data set in this work illustrates the architecture’s ability to learn without the hundreds of thousands or millions of molecules required in previous works. The lower precision values may not be suitable if a small, final working set of molecules is needed. Of course, the choice remains with the user based on the range of model priorities previously discussed. If the retention of favorably binding molecules were crucial alongside a small/medium-sized, initial data set, this protocol would be well-suited to the task. On the other hand, given an ultralarge, initial data set, an iterative protocol, such as that used and developed by Gentile et al.^18,19^ combined with the proposed GCN-ANN architecture would likely be the best option. By iteratively reducing the data set with the dynamic training workflow presented in that work, mean binding affinity would continue to become more negative, leading to extremely high data set enrichment for ultralarge data sets. This necessitates further experimentation; however, this would be a good first step in developing a workflow using the architecture presented in this work.

In summary, this model offers laboratories and organizations the ability to conduct virtual, molecular screening without a lot of resources in terms of cost and time inherent with the traditional processes. The architecture presented in the current study provides a practical tool for screening variable-sized molecular databases. The novelty of the present algorithm lies in the speed of the training and tuning of its models before testing and validating its predictive capabilities alongside novelties presented in the connection of a fingerprint-generating graph convolutional model and a regularized deep neural network for classification. Additionally, this model is capable of mapping similar chemical spaces by using a smaller data set of molecules as compared to contemporary algorithms and hence can be considered as a significant step forward in the field of machine learning-embedded computational drug discovery.

## Data Availability

The code developed for the presented model is available at https://github.com/rivmons/nfp-docking. Data used is available throughout the manuscript text.

## Supporting Information Available

Table with an assessment of the GCN-ANN architecture on the CHEMBL data for AChE and PTP1B using a distribution of actives (<10,000 nM IC50) and inactives; Figure of permutation feature importance analysis, demonstrating how much the GCN-ANN model relies on a particular atomic feature for targets (PDF)
