# Supplementary material for: Efficient and Explainable Virtual Screening of Molecules through Fingerprint-Generating Networks Integrated with Artificial Neural Networks
Source: ACS Omega. 2025 Jan 28;10(5):4896–911. doi: 10.1021/acsomega.4c10289 (PMC11822703; doi:10.1021/acsomega.4c10289)
Supplement: Supplementary file 1 — ao4c10289_si_001.pdf [file ao4c10289_si_001.pdf]

Supporting Information

Efficient and Explainable Virtual Screening of Molecules Through Fingerprint-Generating Networks Integrated with Artificial Neural Networks

Rivaaj Monsia and Sudeep Bhattacharyya*

Department of Chemistry and Biochemistry, University of Wisconsin- Eau Claire, Eau Claire, WI, 54701, USA.

*To whom correspondence should be addressed: phone, (715) 836-2278; fax, (715) 836-4979; e-mail, bhattas@uwec.edu

Table S1. Assessment of the GCN-ANN architecture on the CHEMBL data for AChE and PTP1B using a distribution of actives (< 10000 nM IC50) and inactives. These datasets were split 70-15-15 train-test-validation and the evaluation metrics on the test dataset are provided after training and validation using the proposed GCN-ANN model.

Evaluation metrics/ target enzymes	Actives	Inactives	Recall	Precision	ROC-AUC	PR-AUC	F1 score
AChE	4405	3295	0.97	0.67	0.84	0.87	0.80
PTP1B	1935	1993	0.95	0.62	0.83	0.83	0.75

Figure S1. Permutation feature importance, demonstrating how much the GCN-ANN model relies on a particular atomic feature for targets: a) AChE and GST; b) PAP and PTP1B; c) NQO1 and NQO2. The horizontal axis (in percentage points) measures the difference in accuracy between the original feature matrix and the feature matrix with a specific, permuted feature vector. A higher positive value indicates a larger decrease in accuracy from the original feature vector to the permuted feature vector. The provided data is based on 5 different trials for each permutation experiment such that a mean difference in accuracy and associated standard deviation can be calculated.

a)

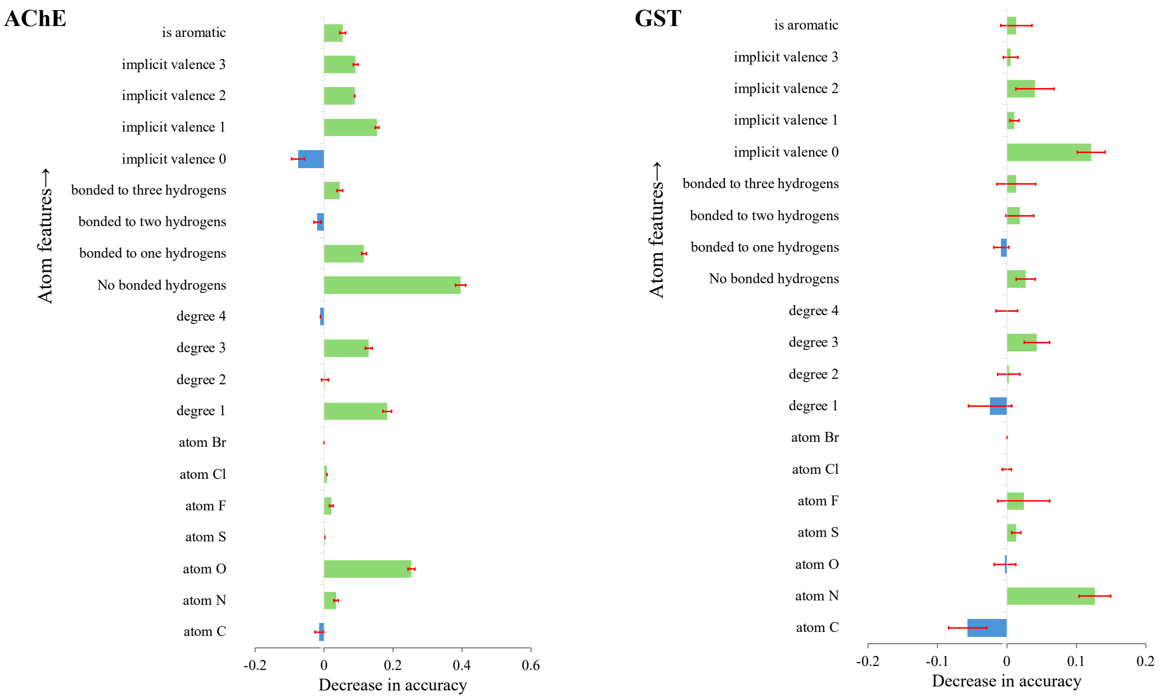

b)

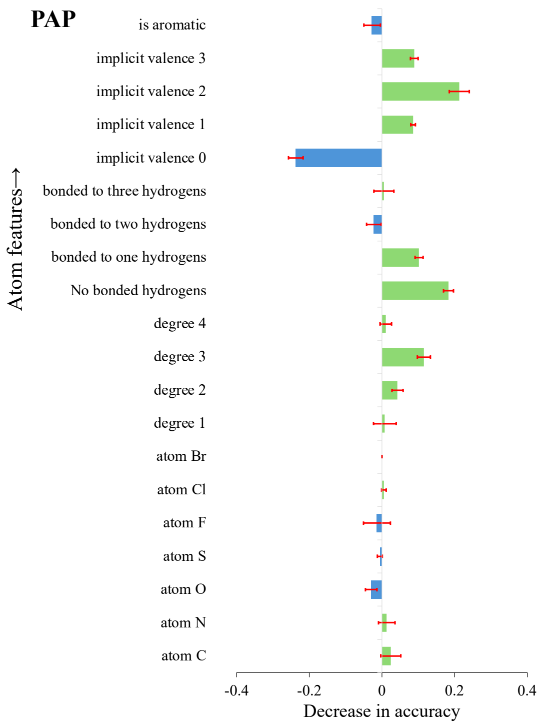
PAP
is aromatic
implicit valence 3
implicit valence 2
implicit valence 1
implicit valence 0
bonded to three hydrogens
bonded to two hydrogens
bonded to one hydrogens
No bonded hydrogens
degree 4
degree 3
degree 2
degree 1
atom Br
atom Cl
atom F
atom S
atom O
atom N
atom C
Atom features⟶
-0.4
-0.2
0
0.2
0.4
Decrease in accuracy

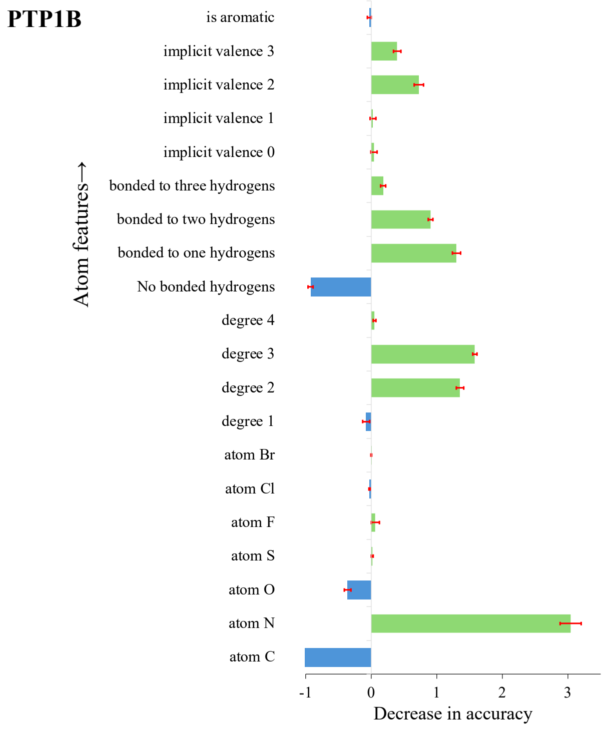
PTP1B
is aromatic
implicit valence 3
implicit valence 2
implicit valence 1
implicit valence 0
bonded to three hydrogens
bonded to two hydrogens
bonded to one hydrogens
No bonded hydrogens
degree 4
degree 3
degree 2
degree 1
atom Br
atom Cl
atom F
atom S
atom O
atom N
atom C
Atom features⟶
-1
0
1
2
3
Decrease in accuracy

c)

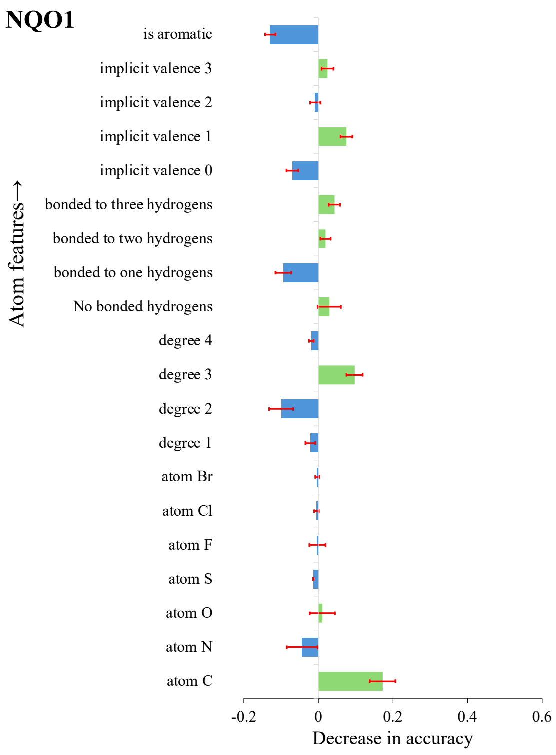
NQO1
is aromatic
implicit valence 3
implicit valence 2
implicit valence 1
implicit valence 0
bonded to three hydrogens
bonded to two hydrogens
bonded to one hydrogens
No bonded hydrogens
degree 4
degree 3
degree 2
degree 1
atom Br
atom Cl
atom F
atom S
atom O
atom N
atom C
Atom features⟶
-0.2
0
0.2
0.4
0.6
Decrease in accuracy

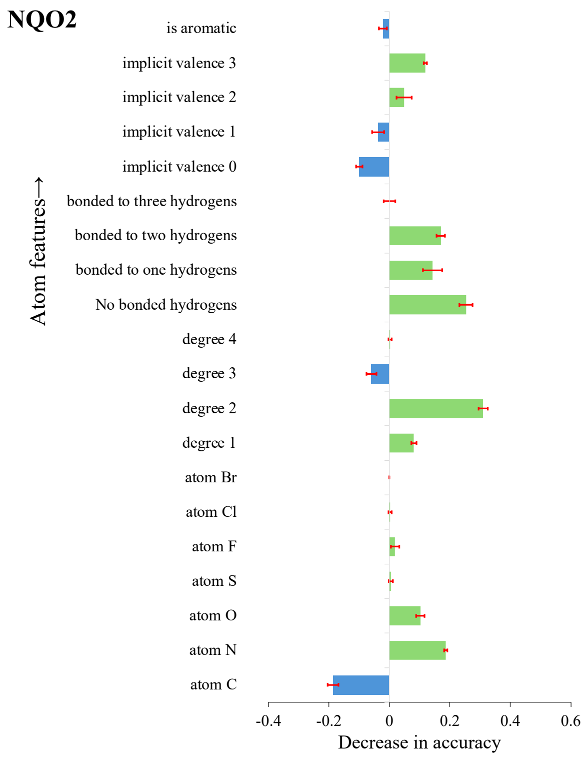
NQO2
is aromatic
implicit valence 3
implicit valence 2
implicit valence 1
implicit valence 0
bonded to three hydrogens
bonded to two hydrogens
bonded to one hydrogens
No bonded hydrogens
degree 4
degree 3
degree 2
degree 1
atom Br
atom Cl
atom F
atom S
atom O
atom N
atom C
Atom features⟶
-0.4
-0.2
0
0.2
0.4
0.6
Decrease in accuracy
